# Transcriptional markers of sub-optimal nutrition in developing *Apis mellifera* nurse workers

**DOI:** 10.1186/1471-2164-15-134

**Published:** 2014-02-15

**Authors:** Vanessa Corby-Harris, Beryl M Jones, Alexander Walton, Melissa R Schwan, Kirk E Anderson

**Affiliations:** 1 Carl Hayden Bee Research Center, USDA Agricultural Research Service, 2000 E. Allen Road, Tucson, Arizona 85719, USA; 2 Department of Entomology, University of Arizona, Tucson, Arizona 85721, USA; 3 Current address: Program in Ecology, Evolution, and Conservation Biology, University of Illinois at Urbana-Champaign, Urbana, IL 61801, USA; 4 Current address: Department of Ecology, Evolution, and Organismal Biology, Iowa State University, Ames, IA 50011, USA

**Keywords:** Transcriptome, *Apis mellifera*, Nutrition, Starvation, Nurse, Development, Physiology

## Abstract

**Background:**

Honey bees (*Apis mellifera*) contribute substantially to the worldwide economy and ecosystem health as pollinators. Pollen is essential to the bee’s diet, providing protein, lipids, and micronutrients. The dramatic shifts in physiology, anatomy, and behavior that accompany normal worker development are highly plastic and recent work demonstrates that development, particularly the transition from nurse to foraging roles, is greatly impacted by diet. However, the role that diet plays in the developmental transition of newly eclosed bees to nurse workers is poorly understood. To further understand honey bee nutrition and the role of diet in nurse development, we used a high-throughput screen of the transcriptome of 3 day and 8 day old worker bees fed either honey and stored pollen (rich diet) or honey alone (poor diet) within the hive. We employed a three factor (age, diet, age x diet) analysis of the transcriptome to determine whether diet affected nurse worker physiology and whether poor diet altered the developmental processes normally associated with aging.

**Results:**

Substantial changes in gene expression occurred due to starvation. Diet-induced changes in gene transcription occurring in younger bees were largely a subset of those occurring in older bees, but certain signatures of starvation were only evident 8 day old workers. Of the 18,542 annotated transcripts in the *A. mellifera* genome, 150 transcripts exhibited differential expression due to poor diet at 3d of age compared with 17,226 transcripts that differed due to poor diet at 8d of age, and poor diet caused more frequent down-regulation of gene expression in younger bees compared to older bees. In addition, the age-related physiological changes that accompanied early adult development differed due to the diet these young adult bees were fed. More frequent down-regulation of gene expression was observed in developing bees fed a poor diet compared to those fed an adequate diet. Functional analyses also suggest that the physiological and developmental processes occurring in well-fed bees are vastly different than those occurring in pollen deprived bees. Our data support the hypothesis that poor diet causes normal age-related development to go awry.

**Conclusion:**

Poor nutrition has major consequences for the expression of genes underlying the physiology and age-related development of nurse worker bees. More work is certainly needed to fully understand the consequences of starvation and the complex biology of nutrition and development in this system, but the genes identified in the present study provide a starting point for understanding the consequences of poor diet and for mitigating the economic costs of colony starvation.

## Background

The European honey bee (*Apis mellifera*) contributes substantially to the worldwide economy and ecosystem health by pollinating a wide variety of plants [1]. Honey bee colonies function as a unit or superorganism, where facultatively sterile female workers support the queen and her developing brood through a progression of tasks under temporal, genetic, environmental, and physiological control [2,3]. Age is intimately tied to development, and shifts in worker behavior over time are accompanied by dramatic physiological, anatomical, and physiological changes [4-6]. Despite the dramatic nature of these shifts, this network is flexible, where behaviors can accelerate, slow, or revert in response to the changing needs of the colony, such as in times of stress or changes in colony demography [3,7].

Pollen is essential to the diet of larvae and young adult workers [8,9]. Larvae acquire protein through worker jelly, a rich secretion from nurse workers’ hypopharyngeal glands (HGs). After eclosion, very young worker bees (less than ~3 days of age) receive a diet of regurgitated pollen and worker jelly [2]. Between 3 and 8 days of age, workers transition to a diet of beebread, stored pollen mixed with sugars and microbes [2,10,11]. Much of the developing adults’ protein, lipids, and micronutrients come from beebread, and these nutrients stimulate the growth of HGs and the fat body (reviewed in [10]) in preparation for the physiologically demanding nurse (~8-14 days) and forager (~14-21 days) roles. Nurses support developing brood and young adults through their HG secretions, and foragers rely primarily on nutrient stores to supplement the small amount of nutrients consumed via trophallaxis and while foraging for nectar, water, and pollen to support the hive [10,12]. Worker pollen consumption increases until nursing age and then subsequently decreases (reviewed in [10]), so the protein assimilation that precedes nursing is critical to colony growth and ontogeny.

Managed honey bees experience long periods of nutritional stress. The majority of honey bees in the U.S. are managed by migratory beekeepers whose livelihood depends on moving hundreds to thousands of colonies to crops requiring pollination. Spring buildup is a crucial time when honey bee colonies that have survived the winter are prepared for pollination of the first crop of the season. Colonies typically come out of winter with little to no pollen stores because any stores from the previous summer and fall were used to sustain the colony through the harsh winter. During spring buildup or throughout the pollination season beekeepers supplement their hives with homemade combinations of protein, sugars, micronutrients, phagostimulants, and antimicrobials that are an incomplete replacement for natural pollen [10,13]. Commercially available pollen substitutes are also used and bees consume and colonies grow in response to these substitutes as well as patties containing natural pollen [14,15]. Nonetheless, recent surveys of beekeepers in small and large commercial operations rank starvation as a major cause of colony loss [16,17], and so there is still a great need for understanding the basic biology of starvation and for developing markers to assess the sublethal effects of poor diet in young adult honey bees at this critical life stage.

Two recent studies on bees kept in cages away from their hive illustrate the utility of high-throughput methods such as oligonucleotide microarrays and messenger RNA sequencing (mRNA-Seq) for studying the biological signatures of dietary stress in nurse worker honey bees. Using mRNA-Seq in whole abdomens (including the digestive tract), Alaux *et al*. [18] found that metabolic pathways involved in nutrient-sensing, metabolism, aging, and immunity were up-regulated in nurses fed pollen, while pathways involved in stress response and the regulation of gene expression were down-regulated. Ament *et al*. [19] used oligonucleotide microarrays to study gene expression in the abdominal carcasses (excluding the digestive tract) of nurses and foragers and found that pollen intake caused the activation of nutrient metabolism processes and reduced expression of transcripts involved in glycolysis, growth and development, neurogenesis, reproduction, and muscle contraction. These two studies used different approaches, yet both find that genes controlling transcriptional regulation and cell signaling are down-regulated in bees fed pollen while those controlling biosynthesis and lipid metabolism are up-regulated ([18,19] and see Additional file 1: Table S1, Additional file 2: Table S2 and Additional file 3: Figure S1). However, it is still unclear (1) whether these responses apply to bees in a natural hive setting and (2) how these signatures of incipient starvation accompany the developmental and physiological changes that occur with age. More work must be done to understand how young adult bees respond to poor diet and to understand the basic biology of nutrition in this system.

We aimed to further understand the transcriptional changes associated with starvation during early adult development. To this end, we assayed 3 day old and 8 day old bees kept in normally functioning hives and caged over only honey (poor diet) or honey and bee bread (rich diet) and allowed trophallaxis with the rest of their colony. Whole transcriptomes from abdominal carcasses (i.e., the fat body, the reduced reproductive organs, exoskeleton, trachea, and muscles) were analyzed to focus on changes in the *A. mellifera* abdomen exclusive of the digestive tract. We hypothesized that (1) starvation and aging would greatly impact gene expression and physiology, (2) that starvation would impact young bees differently than older bees, and that (3) early adult development (aging) would be affected by diet. We also aimed to provide testable predictions for research on development and nutrition in *A. mellifera* and develop targets for improving the productivity of bee colonies under nutritional stress. Here, we report that starvation greatly affects the expression of genes related to nurse physiology and development and that the processes that occur as adults develop into nurse bees are altered by sub-optimal diet.

## Results

### Bees fed pollen had larger hypopharyngeal glands (HG) compared to those that were not fed pollen

**Figure 1:**
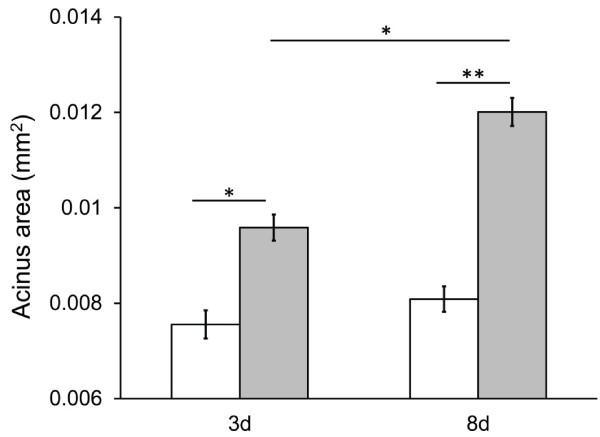
**Hypopharyngeal gland (HG) sizes of nurse workers fed or deprived of pollen.** HG size was significantly affected by both diet and age in developing workers fed a diet of pollen and honey (grey bars) or a diet of honey alone (white bars), as measured by HG acinus size (mm^2^). Error bars represent standard error for the mean acinus size across the three colonies (*N* = 3) tested. Asterisks represent the results of a post-hoc Tukey-Kramer test on the mean acinus sizes for each diet by treatment combination and represent either a *p* < 0.05 (*) or *p* < 0.001 (**) level of significance. There was no significant difference in mean acinus size between 3d and 8d old bees fed only honey.

We began by assessing whether bees that were fed only honey (no pollen) had reduced hypopharyngeal glands, a classic signature of starvation. Hypopharyngeal gland (HG) size was measured in twelve classes of bees (2 ages × 2 diets × 3 host colonies). For each of these twelve treatment combinations, HGs were dissected from approximately 5 bees and 10 randomly selected acini were measured for each gland. Hypopharyngeal gland (HG) size was significantly affected by all factors tested in the model – diet (F_1,4_=160.22, p=0.0002), host colony (i.e., the host colony that the focal bees were placed into; F_2,8_=13.10, p=0.0030), age (F_1,4_=39.50, p=0.0033), diet by colony (F_2,8_=96.80, p<0.0001), age by diet (F_1,4_=15.97, p=0.0162), age by colony (F_2,8_=35.89, p=0.0001), and the three-way age by diet by colony interaction (F_2,4_=13.61, p=0.0164). *Post-hoc* analyses showed significant differences between 3d old bees fed the rich versus poor diet, 8d old bees fed the rich versus poor diet, and 3d versus 8d bees fed the rich diet, but no difference between 3d and 8d old bees fed only honey (Figure 1).

Due to the significant interaction between diet and colony in the full model discussed above, the effect of diet and age on HG size was also investigated for each colony separately. For colony A, the effect of diet (F_1,4_=217.65, p=0.0001) and the interaction between age and diet (F_1,2_=34.25, p=0.0280) were significant but the effect of age (F_1,4_=6.80, p=0.0596) was not (Additional file 4: Figure S2). For colony B, the effect of diet (F_1,4_=189.38, p=0.0002) and the interaction between age and diet (F_1,2_=14.05, p=0.0200) were significant but the effect of age (F_1,4_=2.85, p=0.1667) was not (Additional file 4: Figure S2). For colony C, the effect of age (F_1,4_=72.60, p=0.0010) and diet (F_1,4_=26.49, p=0.0068) were significant, but the effect of the age × diet interaction (F_1,2_=0.50, p=0.5511) was not (Additional file 4: Figure S2).

The HG sizes of bees that were used to evaluate the predictive power of the mRNA-Seq results with qPCR (but that were not part of the mRNA sequencing experiment), were also measured. Only 8d old bees were used in this phase of the experiment, yielding 6 different classes of bees (2 diets × 3 colonies). For each of these 6 different treatment combinations, the HGs of 5 bees were dissected and 10 randomly selected acini were measured for each gland. The HGs from 8d old bees fed pollen were significantly larger than those from 8d old bees fed only honey (8d rich diet mean acinus area: 0.015 mm^2^±0.002 s.e., 8d poor diet mean acinus area: 0.007 mm^2^±0.0003 s.e.; F_2,4_=24.48, p=0.0057).

### Sequencing statistics and broad patterns

Across all twelve libraries (2 ages × 2 diets × 3 host colonies), approximately 149.4 million paired end reads passed the initial quality control filters and approximately 103 million paired end reads mapped to the *A. mellifera* genome (Additional file 5: Table S3). Across the twelve libraries, an average of 8.58 million (67.5%) paired end reads mapped to the *A. mellifera* genome per library. In total, the expression of 67,002 exons (non-singleton exons; see Methods) mapping to 12,340 transcripts (containing ≥2 exons; see Methods) was differentially expressed (Additional file 6: Table S4 [20]). The interaction between diet and age did not significantly impact the expression of any exons, and so results are presented below for exons and transcripts that changed due to the main effect of diet, the main effect of age, the effect of diet at either 3d or 8d, or the effect of age in bees fed a rich or poor diet.

### Starvation caused drastic differences in gene expression, and these differences are most evident when the effect of starvation is assessed for each age class separately

We investigated whether starvation impacted gene expression by first testing whether transcription was affected by the main effect of diet (i.e., exhibited consistent differences due to diet in both 3d and 8d bees). The expression of 24 exons mapping to 13 transcripts was significantly affected by the main effect of diet and all were up-regulated in bees fed pollen compared to those that were fed a poor diet (Table 1). These 24 up-regulated exons, which included transcripts encoding vitellogenin and worker-enriched antennal transcript, mapped to 5 orthologues (Additional file 2: Table S2 [20]) but were not associated with any biological processes or gene annotation clusters.

**Table 1:** Differentially expressed exons, transcripts, and orthologues for each category investigated

	**Diet**						**Age**						**Average**
	**↑ Rich**			**↑ Poor**			**↑ Young**			**↑ Old**			
	**Main**	**3d**	**8d**	**Main**	**3d**	**8d**	**Main**	**Rich diet**	**Poor diet**	**Main**	**Rich diet**	**Poor diet**	
Exons^A^	24	360	42,497	0	26	24,314	187	91	184	76	94	72	
Transcripts^B^	13	150	11,393	0	23	8,983	81	39	77	18	23	14	
Annotated transcripts^C^	11	134	9,642	0	16	7,584	74	33	71	18	21	13	
*A. mellifera* transcripts placed with *D. melanogaster* orthologues^D^	5	59	4,580	-	6	3,569	48	9	49	4	8	3	
Percent of total transcripts identified in the present study with an orthologue^E^	38	39	40	-	26	40	59	23	64	22	35	21	37

^A^Does not contain singletons (see Methods).

^B^Differentially expressed transcripts containing ≥ 2 exons.

^C^Differentially expressed transcripts annotated in GenBank. These values correspond to those presented in Figures 2a and 3a.

^D^These values correspond to those presented in Figures 2b and 3b.

^E^Total number of transcripts includes both the annotated and unannotated transcripts.

The genes that were up- or down-regulated due to starvation in either young (3d old) or old (8d old) bees were analyzed separately to determine whether the effects of starvation varied with the age of the bee. Starvation caused more frequent down-regulation of transcripts in younger bees (150 out of 173; 78.5%) compared to older bees (11,393 out of 20,376; 55.9%; *X*_*1*_^2^=64.82, *p*<0.0001; Table 2 and Additional file 6: Table S4 [20]). Comparisons using exons instead of genes containing ≥2 exons per transcript yielded similar results (*X*_*1*_^2^=144.83, *p*<0.0001; Additional file 7: Table S5).

**Table 2:** Transcript-based analysis of expression with longer periods of starvation or increasing age

**Factor**	**Constant**	**Transcripts changed**^**A**^	**Increased**^**A**^	**Decreased**^**A**^	**% Transcripts decreased**^**B**^	**Difference in % decrease?**	
						***Χ***^**2**^	***p***
Starvation	3d	173	23	150	78.5	64.82	<0.0001
	8d	20,376	8,983	11,393	55.9		
Aging	Poor diet	91	14	77	79.3	8.33	0.0039
	Rich diet	62	23	39	62.9		

^A^Differentially expressed transcripts contain ≥2 exons. Transcripts containing single exons were omitted from the analysis.

^B^Of the transcripts that differed due to each factor combination.

**Figure 2:**
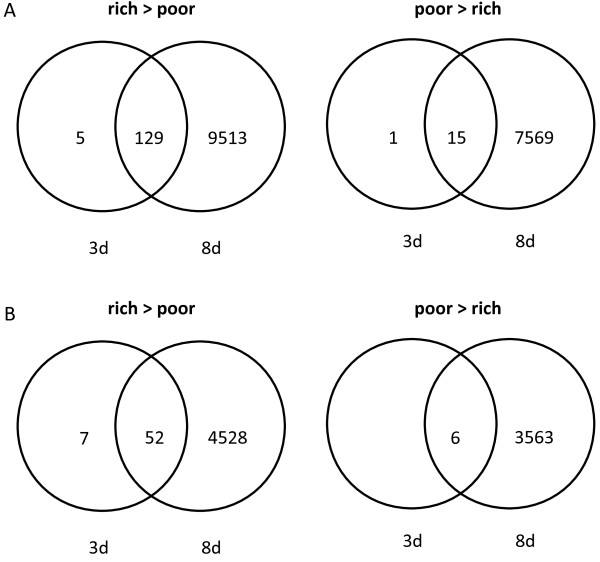
**The impact of diet on bees aged 3 days or 8 days.** The number of **(A)** annotated transcripts and **(B)** orthologues differentially expressed as a function of diet in bees fed a rich or poor diet for 3d or 8d. Panels on the left (rich > poor) represent terms down-regulated with starvation. Panels on the right (poor > rich) represent terms whose expression was higher in bees fed a poor diet.

We determined how patterns of diet-induced gene up- or down-regulation overlapped between the two age classes. Transcripts and orthologues that changed with diet at 3d of age were largely a subset of those that changed at 8d of age (Figure 2). Transcripts down-regulated due to starvation in both 3d and 8d old bees (Figure 2, left side, rich>poor) included those encoding cuticular proteins, apidermins, worker-enriched antennal transcript, and nitric oxide synthase (Additional file 6: Table S4 [20]) and were associated with chitin metabolism, response to oxidative stress, and motor function (Table 3). Transcripts up-regulated in pollen deprived bees at both time points (Figure 2, right side, poor>rich) included those encoding a histone-arginine methyltransferase, dynein heavy chain, dual oxidase (Duox), and argonaute (Additional file 6: Table S4 [20]) but were not significantly related to any biological processes or gene annotation clusters.

**Table 3:** Biological process (BP) terms and annotation clusters down-regulated in pollen deprived bees at 3d and 8d

**BP term or cluster**	**Description**	**N**	***X***^**2**^_**adj**_^**A**^	**Orthologues**^**B**^
GO:0007155	Cell adhesion (BP)^C^	6	<0.0001	FBgn0034660, FBgn0000547, FBgn0031879, FBgn0000464, FBgn0000117, FBgn0026562
GO:0007155, GO:0008360	Cell adhesion (BP)^C^, regulation of cell shape (BP)^C^	3	<0.0001	FBgn0000547, FBgn0000464, FBgn0000117
GO:0007391	Dorsal closure (BP)^C^	3	0.0037	FBgn0000299, FBgn0000547, FBgn0000117
GO:0046331	Lateral inhibition (BP)^C^	4	0.0059	FBgn0010473, FBgn0000547, FBgn0243514, FBgn0053196
IPR002172	Low-density lipoprotein (LDL) receptor class A repeat	3	<0.0001	FBgn0031879, FBgn0053196, FBgn0261341
IPR013032	EGF-like region, conserved site	5	<0.0001	FBgn0003137, FBgn0026403, FBgn0031879, FBgn0243514, FBgn0053196
GO:0005509	Calcium ion binding (MF)	7	<0.0001	FBgn0026403, FBgn0031879, FBgn0000079, FBgn0031464, FBgn0037963, FBgn0053196, FBgn0026562
IPR003599, IPR007110	Immunoglobulin subtype, Immunoglobulin-like 6	6	<0.0001	FBgn0003137, FBgn0010473, FBgn0000547, FBgn0005666, FBgn0000464, FBgn0004369
IPR003599, IPR007110, IPR003961, IPR008957	Immunoglobulin subtype, Immunoglobulin-like, Fibronectin, type III, Fibronectin type III domain	5	<0.0001	FBgn0010473, FBgn0000547, FBgn0005666, FBgn0000464, FBgn0004369
IPR001660, IPR010993, IPR011510, IPR021129	Sterile alpha motif domain, Sterile alpha motif homology, Sterile alpha motif, type 2, Sterile alpha motif, type 1	3	<0.0001	FBgn0000182, FBgn0031762, FBgn0039075
IPR018247	EF-Hand 1, calcium-binding site	3	0.0019	FBgn0031464, FBgn0013809, FBgn0026562
GO:0004867	Serine-type endopeptidase inhibitor activity (MF)	3	<0.0001	FBgn0003137, FBgn0026721, FBgn0053196
GO:0006030, GO:0008061, IPR002557	Chitin metabolic process (BP)^C^, chitin binding (MF), Chitin binding domain	3	<0.0001	FBgn0027600, FBgn0051973, FBgn0261341
IPR017853	Glycoside hydrolase, superfamily	3	<0.0001	FBgn0053080, FBgn0000079, FBgn0026415
GO:0030036	Actin cytoskeleton organization^D^	2	0.0080	FBgn0000182, FBgn0085447
GO:0006470	Protein dephosphorylation^D^	2	0.0242	FBgn0000464, FBgn0004369
GO:0007498	Mesoderm development^D^	2	0.0130	FBgn0005666, FBgn0026562
GO:0008045	Motor axon guidance^D^	2	0.0013	FBgn0000464, FBgn0004369
GO:0005975	Carbohydrate metabolic process^D^	2	0.0008	FBgn0053080, FBgn0000079
GO:0007414	Axonal defasciculation^D^	2	<0.0001	FBgn0010473, FBgn0004369
GO:0007156	Homophilic cell adhesion^D^	2	0.0001	FBgn0000547, FBgn0037963
GO:0006979	Response to oxidative stress^D^	2	0.0033	FBgn0031464, FBgn0036756
GO:0016337	Cell-cell adhesion^D^	2	<0.0001	FBgn0000547, FBgn0000117

^A^*X*^2^_adj_ refers to the significance of the Fisher’s exact test with a Benjamini-Hochberg correction for multiple comparisons.

^B^Orthologues correspond to those pictured in Figure 2B (left panel) and represent *Drosophila melanogaster* genes that are listed in FlyBase.

^C^Terms that were significant BP terms and were part of a significant gene annotation cluster.

^D^Terms that represented significant BP terms but did not form a significant gene annotation cluster.

The genes were down-regulated upon starvation exclusively at 3d or 8d of age (Figure 2, left side, rich>poor) were assessed to determine whether the processes associated with starvation differed in young versus old bees. Transcripts down-regulated due to starvation in young (3d old) but not old (8d old) bees included apidermin 3, cuticular proteins, and ecdysis triggering hormone (Additional file 6: Table S4 [20]) but no biological processes or annotation clusters. Transcripts down-regulated in pollen deprived bees at 8d but not at 3d included glutathione S-transferases, major royal jelly (MRJP) proteins 1 through 9, hexamerins, DNA methyltransferases, cytochrome p450s, and immune genes (Additional file 6: Tables S4 [20]) but were not associated with any biological processes or gene annotation clusters.

Genes that were up-regulated upon starvation exclusively at 3d or 8d of age were next assessed (Figure 2, right side, poor>rich) to determine whether the increases in gene expression that occurred differed in young and old bees. One transcript encoding transient receptor potential-gamma protein-like (XM_001122469) was up-regulated in pollen deprived bees exclusively at 3d (Additional file 6: Tables S4 [20]) but this transcript was not orthologous to a *D. melanogaster* gene. Transcripts up-regulated in pollen deprived bees exclusively at 8d included immune recognition genes, cytochrome P450s, cuticular proteins, MRJPs, hexamerins, DNA methyltransferases, and one microRNA (Additional file 6: Tables S4 [20]) but were not associated with any biological processes or annotation clusters.

### Starvation alters normal age-associated nurse development

To ask whether certain signatures of early adult development were consistently expressed in the same direction and in similar magnitude for well-fed and underfed workers, we determined whether the main effect of age significantly impacted gene expression. A total of 263 exons (mapping to 99 transcripts) were differentially expressed due to the main effect of age (Table 1). 187 (71%) of the exons (mapping to 81 transcripts) were down-regulated and 76 (29%) of the exons (mapping to 18 transcripts) were up-regulated as bees as they aged from 3d to 8d old (Table 1). The transcripts down-regulated with age included those coding for vitellogenin, tetraspanin 6, and AncR-1 non-coding nuclear RNA (Additional file 6: Table S4 [20]) and corresponded to 48 orthologues associated with transcriptional regulation (Table 4). The transcripts up-regulated with age included hexamerins, immune genes, juvenile hormone esterase, and odorant binding proteins (Additional file 6: Table S4 [20]) but were not associated with any biological processes or annotation clusters.

**Table 4:** Biological process (BP) terms and annotation clusters significantly down-regulated due to the main effect of age

**Cluster**	**Description**	**N**	***X***^**2**^_**adj**_^**A**^	**Orthologues**^**B**^
GO:0005524	ATP binding (MF)	10	0.0029	FBgn0260990, FBgn0261014, FBgn0032243, FBgn0025743, FBgn0026059, FBgn0000723, FBgn0030343, FBgn0015615, FBgn0036486, FBgn0051729
Kegg:03040	Spliceosome	3	0.0099	FBgn0031390, FBgn0038927, FBgn0031266
GO:0007391	Dorsal closure (BP)^C^	3	0.0048	FBgn0010434, FBgn0000723, FBgn0044323
IPR001680, IPR011046, IPR019775	WD40 repeat, WD40 repeat-like-containing domain, WD40 repeat, conserved site	5	<0.0001	FBgn0037758, FBgn0029903, FBgn0044323, FBgn0038927, FBgn0043362
IPR001680, IPR011046, IPR019775, IPR020472, IPR019782, IPR017986, IPR019781	WD40 repeat, WD40 repeat-like-containing domain, WD40 repeat, conserved site, G-protein beta WD-40 repeat, WD40 repeat 2, WD40-repeat-containing domain, WD40 repeat, subgroup	4	<0.0001	FBgn0029903, FBgn0044323, FBgn0038927, FBgn0043362
IPR011046	WD40 repeat-like-containing domain	6	<0.0001	FBgn0037758, FBgn0029903, FBgn0024698, FBgn0044323, FBgn0038927, FBgn0043362
GO:0003779	Actin binding (MF)	3	0.0109	FBgn0035347, FBgn0010434, FBgn0029903
IPR016024	Armadillo-type fold	4	0.0248	FBgn0260990, FBgn0030674, FBgn0043362, FBgn0031266
GO:0016319	Mushroom body development^D^	2	0.0071	FBgn0025743, FBgn0010051
GO:0007030	Golgi organization^D^	2	0.0019	FBgn0261014, FBgn0033075

^A^*X*^2^_adj_ refers to the significance of the Fisher’s exact test with a Benjamini-Hochberg correction for multiple comparisons.

^B^Orthologues represent *Drosophila melanogaster* genes similar to the differentially expressed transcripts discovered in the present study that are listed in FlyBase.

^C^Terms that were significant BP terms and were part of a significant gene annotation cluster.

^D^Terms that represented significant BP terms but did not form a significant gene annotation cluster.

Transcripts that were up- or down-regulated with age were investigated in pollen deprived and well-fed bees separately to determine whether the signatures of normal age-associated development differed in underfed versus well-fed bees. Transcript expression decreased with age more frequently in bees fed a poor diet (77 out of 91; 79.3%) compared to bees fed a rich diet (39 out of 62; 62.9%; *X*^2^_1_=8.33, *p*=0.0039; Table 2 and Additional file 6: Table S4 [20]). Exon-based analyses also supported this trend: 71.9% of exons were down-regulated with age in pollen deprived bees compared to the 49.2% down-regulated with age in bees fed pollen (*X*^2^_1_=22.59, *p*<0.0001; Additional file 7: Table S5).

The overlap between transcripts differentially regulated due to age in bees fed either diet was investigated to determine what processes that were up- or down-regulated with age were common between underfed and well-fed bees. Only one transcript (with no orthologue), encoding a hypothetical protein (Additional file 6: Table S4 [20]), was down-regulated with age in bees fed both diets. Transcripts up-regulated with age in bees fed either diet included apidaecin, cytochrome P450 342A1, and hexamerin 110 (Additional file 6: Table S4 [20]). These transcripts were associated with one orthologue that was not associated with a biological process or annotation cluster.

**Figure 3:**
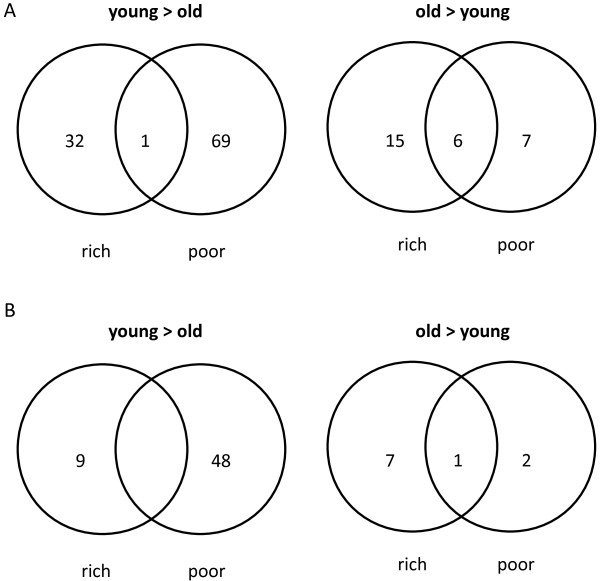
**The impact of increasing age for bees fed a rich or poor diet.** The number of **(A)** annotated transcripts and **(B)** orthologues differentially expressed as a function of age in bees fed a rich or poor diet. Panels on the left (young > old) represent terms down-regulated with age in bees fed either a rich or poor diet. Panels on the right (old > young) represent terms whose expression was up-regulated with age in bees fed either a rich or poor diet.

The transcripts down-regulated with age in either pollen deprived or well-fed bees were investigated (Figure 3, left panels, young>old) to determine how age-associated decreases in gene expression differed with diet. Transcripts down-regulated in older bees fed only the rich diet included apidermin and worker anntenal transcript (Additional file 6: Table S4 [20]) and these transcripts were involved in open tracheal system development and chitin metabolism (Table 5). Transcripts down-regulated with age in bees fed only the poor diet included AncR-1 non-coding nuclear RNA (Ancr-1) and tetraspanin 6 (Additional file 6: Table S4 [20]) and were involved in transcription, development, sensory system development, and mRNA splicing (Table 6).

**Table 5:** Biological process (BP) terms down-regulated with age only in bees fed the rich diet

**BP term**	**Description**	**N**	***Χ***^**2**^_**adj**_^**A**^	**Orthologues**^**B**^
GO:0007424	Open tracheal system development	2	<0.0001	FBgn0260653, FBgn0261341
GO:0006030	Chitin metabolic process	3	<0.0001	FBgn0051973, FBgn0260653, FBgn0261341
GO:0035159	Regulation of tube length, open tracheal system	2	<0.0001	FBgn0260653, FBgn0261341

^A^*X*^2^_adj_ refers to the significance of the Fisher’s exact test with a Benjamini-Hochberg correction for multiple comparisons.

^B^Orthologues correspond to those pictured in Figure 3B (left panel) and represent *Drosophila melanogaster* genes that are listed in FlyBase.

**Table 6:** Biological process (BP) terms and annotation clusters down-regulated with age only in pollen deprived bees

**BP term or cluster**	**Description**	**N**	***X***^**2**^_**adj**_^**A**^	**Orthologues**^**B**^
GO:0007391	Dorsal closure (BP)^C^	3	<0.0001	FBgn0044323, FBgn0010434, FBgn0000723
GO:0005488	Binding (MF)	3	0.0357	FBgn0031266, FBgn0021847, FBgn0030674
GO:0046331	Lateral inhibition (BP)^C^	3	0.0075	FBgn0029685, FBgn0015615, FBgn0025743
IPR016024	Armadillo-type fold	4	<0.0001	FBgn0031266, FBgn0043362, FBgn0260990, FBgn0030674
GO:0005524	ATP binding (MF)	9	<0.0001	FBgn0051729, FBgn0036486, FBgn0261014, FBgn0032243, FBgn0015615, FBgn0025743, FBgn0026059, FBgn0260990, FBgn0000723
GO:0005524, GO:0006468, IPR002290, IPR000719	ATP binding (MF),protein phosphorylation (BP), Serine/threonine-protein kinase domain, Protein kinase, catalytic domain	3	0.0080	FBgn0025743, FBgn0260990, FBgn0000723
IPR011046	WD40 repeat-like-containing domain	7	<0.0001	FBgn0024698, FBgn0044323, FBgn0027518, FBgn0043362, FBgn0037758, FBgn0029903, FBgn0038927
GO:0003779	Actin binding (MF)	3	<0.0001	FBgn0010434, FBgn0029903, FBgn0035347
GO:0000398	Nuclear mRNA splicing, via spliceosome (BP)^C^	3	0.0080	FBgn0031266, FBgn0034002, FBgn0038927
Kegg:03040	Spliceosome	3	<0.0001	FBgn0031266, FBgn0038927, FBgn0031390
GO:0006468	Protein phosphorylation^D^	3	0.0148	FBgn0025743, FBgn0260990, FBgn0000723
GO:0006378	mRNA polyadenylation^D^	2	<0.0001	FBgn0024698, FBgn0260780
GO:0016319	Mushroom body development^D^	2	<0.0001	FBgn0010051, FBgn0025743
GO:0048749	Compound eye development^D^	2	0.00488	FBgn0043362, FBgn0025743
GO:0006813	Potassium ion transport^D^	2	<0.0001	FBgn0037244, FBgn0037758
GO:0006281	DNA repair^D^	2	<0.0001	FBgn0036486, FBgn0015283
GO:0007030	Golgi organization^D^	2	<0.0001	FBgn0261014, FBgn0033075

^A^*X*^2^_adj_ refers to the significance of the Fisher’s exact test with a Benjamini-Hochberg correction for multiple comparisons.

^B^Orthologues correspond to those pictured in Figure 3B (left panel) and represent *Drosophila melanogaster* genes that are listed in FlyBase.

^C^Terms that were significant BP terms and were part of a significant gene annotation cluster.

^D^Terms that represented significant BP terms but did not form a significant gene annotation cluster.

Transcripts up-regulated with age in either pollen deprived or well-fed bees were investigated (Figure 3, right panels, young<old) to determine how age-associated increases in expression differed with diet. Transcripts up-regulated in older bees fed only the rich diet included those encoding apolipophorin and peptidoglycan recognition proteins S2 and S3 (Additional file 6: Table S4 [20]) and were associated with immunity and oxidation-reduction (Table 7). Transcripts up-regulated in older bees fed only the poor diet included juvenile hormone esterase, vitellogenin, hexamerin 70a, cytochrome P450 4AZ1 and two odorant binding proteins (Additional file 6: Table S4 [20]). The two orthologues related to these transcripts were not associated with any biological processes or annotation clusters.

**Table 7:** Biological processes up-regulated with age only in bees fed the rich diet

**BP term**	**Description**	**N**	***Χ***^**2**^_**adj**_^**A**^	**Orthologues**^**B**^
GO:0055114	Oxidation-reduction process	2	0.0001	FBgn0014032, FBgn0032945
GO:0045087	Innate immune response	2	<0.0001	FBgn0030310, FBgn0043575
GO:0006952	Defense response	2	<0.0001	FBgn0030310, FBgn0043575
GO:0006955	Immune response	2	<0.0001	FBgn0030310, FBgn0043575
GO:0009253	Peptidoglycan catabolic process	2	<0.0001	FBgn0030310, FBgn0043575

^A^*X*^2^_adj_ refers to the significance of the Fisher’s exact test with a Benjamini-Hochberg correction for multiple comparisons.

^B^Orthologues correspond to those pictured in Figure 3B (right panel) and represent *Drosophila melanogaster* genes that are listed in FlyBase.

### Targeted rt-PCR of differentially expressed genes confirmed the mRNA-sequencing results

**Figure 4:**
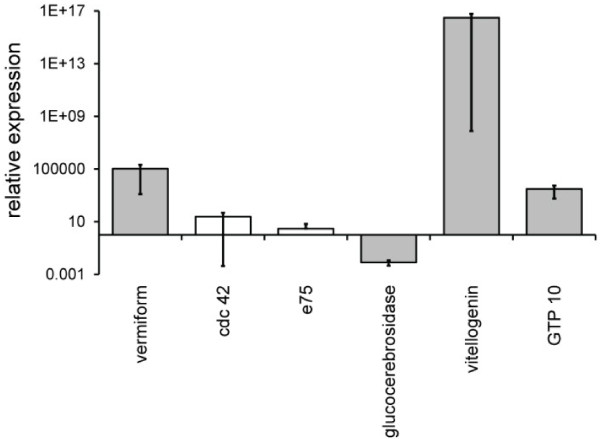
**rt-PCR validation of RNA sequencing results.** The expression of genes in the fat bodies of bees fed a rich diet containing pollen relative to bees fed a poor diet that did not contain pollen is shown. Grey bars indicate significant over-expression (>1) or under-expression (<1) among N = 3 replicate samples using a Mann–Whitney test where the direction of expression agreed with predictions from the mRNA-seq experiment. Expression normalized relative to actin is shown and agreed with the results for expression normalized to GAPDH. White bars indicate results that, on average, agreed with the results of the mRNA-seq experiments but that were non-significant. Error bars represent the standard error of the mean relative expression value.

To verify our results from the mRNA-seq experiments, real-time PCR was used on a small subset of the genes that were differentially regulated according to the mRNA-seq analyses. The expression of 6 transcripts in the abdominal carcass of 8d old bees from three colonies fed honey or honey and bee bread confirmed the mRNA-seq results. The expression of vermiform, vitellogenin, and GTP-binding protein 10 in bees fed a rich diet compared to those fed a poor diet were all greater than one (p=0.049 for each), and the expression of glucocerebrosidase was significantly lower (p=0.049; Figure 4). The expression of Cdc42 and E75 trended upward (>1) in bees fed a rich diet compared to those fed a poor diet, but their relative expression was not significantly different than one (Figure 4). In each case, the direction of expression change agreed with the predictions from the mRNA-sequencing. All rt-PCR results using the GAPDH control were similar to those using the actin control. In sum, the rt-PCR results verified the mRNA-seq results for the small set of transcripts that were tested.

## Discussion

Pollen is a critical component of the diet for young adult bees and can greatly impact their development. Surveys of beekeepers suggest that starvation is a major cause of colony losses and our understanding of how starvation affects bees at the critical young adult stage is incomplete. To understand the basic biology of pollen deprivation and to develop transcriptional markers of starvation in developing nurse worker bees, we used mRNA-seq to measure changes in gene expression in 3d and 8d old bees fed a diet of honey and stored pollen within the hive compared to a restricted diet containing honey but no pollen. Many interesting results became apparent when diet-related transcripts were studied separately for bees aged 3d or 8d and by determining which transcripts were differentially expressed at either 3d or 8d of age. Substantial changes in gene expression were observed when bees were deprived of pollen and these changes were more numerous as bees were deprived of pollen for longer periods of time. Additionally, normal patterns of gene expression that accompanied age-related changes in development were altered in underfed bees. The data suggest that nutrients in pollen regulate the key developmental transitions that honey bees experience during early adult life as they prepare for critical brood care tasks.

Transcripts that were down-regulated in pollen deprived bees at both ages (i.e., due to the model main effect of age) included those with potential roles in development and muscle and motor function. These results agree with those of Ament *et al.*[19], who also found that the transcription of growth, development and muscle contraction genes was reduced in poorly fed nurses. It appears that poor diet is costly to the expression of genes involved in muscle function and growth from a rather early age. Whether this affects the ability of nurse bees to perform in-hive tasks or whether the lasting effects of starvation are evident in foragers remains to be studied. However, it is possible that traits such as foraging ability may be affected by poor diet in early adulthood.

Many more transcripts changed due to starvation in older bees (20,376 transcripts) compared to younger bees (173 transcripts). Of the transcripts where expression changed due to poor diet, there was more frequent down-regulation of gene expression in younger bees (78.5%) compared to older bees (55.9%). Transcriptional changes occurring due to starvation at 3d were largely a subset of those occurring at 8d. Many more diet-related differences were observed only in bees deprived of pollen for 8d, but it is unclear whether the genes differentially expressed exclusively at 8d are so due to the longer period of starvation or because specific processes that are turned on only at later ages are also impacted by diet. The expression of MRJPs, immune genes, and genes encoding detoxification enzymes declined due to poor diet exclusively at later ages. This group of down-regulated transcripts included several members of the CYP4 and CYP6 cytochrome P450 families and one Delta GST, which have roles in insecticide detoxification [21], chitin-binding proteins that maintain the integrity of the peritrophic membrane, which plays a defensive role against chemicals and parasites [22], genes such as PGRP S3 and cytP450 4G11 whose down-regulation is associated with colony collapse disorder CCD; [23], and the trancripts *dicer* and *argonaute* that mediate immunity towards viruses that are sometimes prevalent in failing colonies [24,25]. One potential consequence, therefore, of pollen deprivation in nurses but not newly eclosed bees is reduced resistance to disease and insecticides.

Aging was associated with the overall decline of processes such as development, transcription, and mRNA splicing, and this decline was exacerbated in pollen deprived bees. Age-related transcriptional decline was more frequent in underfed bees (Tables 2 and Additional file 7: Table S5), and the processes occurring as well-fed bees aged were distinct from bees deprived of pollen (Figure 3). Bees fed a rich diet up-regulated genes involved in immunity and down-regulated those involved in tracheal system development and chitin metabolism, possibly as part of a developmental trajectory that prepares them for tasks outside of the hive. Poorly fed bees did not differentially regulate any of the age-related processes occurring in bees fed a normal diet. Pollen deprived bees showed down-regulation of genes involved in transcription and mRNA splicing and a non-coding RNA potentially involved in transcriptional regulation AncR-1; [26]. Loss of transcriptional regulation and the dysregulation of gene expression are associated with age-related functional decline in a variety of taxa ([27-30]; but see [31]). The data therefore suggest that pollen deprivation may cause normal age-related transcriptional regulation to go awry, leading to various downstream effects. For example, aging bees deprived of pollen exhibited the reduced expression of genes involved in mushroom body and compound eye development. If such signals from the fat body impact brain signaling and development, this decrease could be a sign of diet-induced early foraging, as (1) early foraging is associated with nutrient restriction [6], (2) reduced volume of Kenyon cells in the mushroom body is associated with the transition to later age tasks such as foraging [32-34] and (3) the mushroom body is where sensory signals are integrated [35].

Further support for the hypothesis that aging is dysregulated in pollen-deprived bees becomes evident from comparing our results to examples presented in the literature, such as juvenile hormone esterase (JHE; which degrades juvenile hormone), vitellogenin, and hexamerin 70a. Nurses have highest levels of JHE in order to prevent the switch to foraging [36], as high levels of juvenile hormone are associated with the onset of foraging. We observed that for both fed and pollen starved bees JHE expression is higher in 8d old bees compared to 3d old bees. However, the magnitude of this increase was greater in starved bees and this increased expression with age was only significant in starved bees. Vitellogenin is very high in newly emerged workers, decreases slightly at days 1 and 2 post-eclosion, and then peaks at 3 days of age [37]. It then steadily decreases from this highest level until approximately 20 days of age [38,39]. In the present study, vitellogenin expression decreased as expected in well-fed bees aging from 3 days to 8 days old, but the difference was not significant. However, starved bees showed the opposite pattern, with expression increasing as they aged from 3d to 8d, and the change in expression was significant. Cunha *et al*. [40] find that titres of the hexamerin 70a protein decrease a very small amount, if any between emergence and 7d of age. We saw no difference in the expression of hexamerin 70a gene in old versus young bees when they were well-fed. However, when deprived of pollen, expression was significantly lowered as the bees aged. The aberrant expression of these genes in starved but not well-fed bees again suggests that aging is dysregulated in starved bees.

The expression of relatively few genes changed with respect to the main effect of age, yet there was measurable overlap in the patterns of expression when we compared the genes that were up- or down-regulated with age in the poor versus rich diets (for each diet analyzed separately; Figure 3). The transcripts that differed in expression due to the main effect of age showed similar patterns of significant up- or down-regulation for both diets and the magnitude of this change was the same for the different diets. Because we were interested in comparing the aging process in starved versus well-fed bees, we took the additional approach of determining what genes were differentially expressed due to age considering each diet separately. Figure 3 shows the overlap between diets in the transcripts that changed with age and this change was in the same direction for each of the diets. The main reason that these genes were not also significantly affected by the main effect of age was that the magnitude of the age-related change differed between diets, particularly in genes that were down-regulated as bees aged (Additional file 8: Figure S3). This difference in the magnitude of the age-related change in expression that is diet-dependent provides additional support for our hypothesis that the aging process itself differs due to diet.

**Figure 5:**
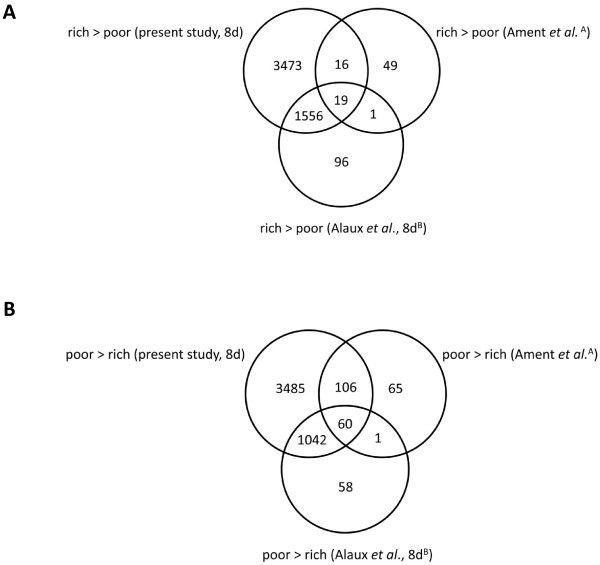
**The core set of biological process impacted by diet.** For transcripts that differed with diet, Venn Diagrams were constructed using the biological process gene ontology (GO) terms presented by Ament *et al*. [19], that were associated with the orthologues found in the present study for the 8d old bees only, and the BeeBase IDs presented by Alaux *et al*. [18]. Terms that were up-regulated in bees fed pollen are presented in panel **A**. Terms that were up-regulated in starved bees are presented in panel **B**. The superscript A indicates that diet-associated genes were identified in the abdominal carcasses for both nurses and foragers in Ament *et al*. [19], and the superscript B indicates that the entire abdomen (including the digestive tract) was utilized in Alaux *et al*. [18]. The data shown from the present study represent terms that were differentially expressed in abdominal carcasses of 8d old bees. 3d old bees were not included in the analysis.

Recent results from Ament *et al*. [19] and Alaux *et al*. [18] illustrate the utility of high-throughput analyses of the honey bee transcriptome for studying the benefits of good nutrition in honey bees. We can relate our results to those of these two studies by comparing the biological process GO terms differentially expressed in the present experiment, the terms found by Ament *et al*. [19], and the terms associated with the differentially expressed genes in Alaux *et al*. [18]. This *post hoc* analysis reveals a core group of biological processes that are up- or down-regulated in the abdomens of young pollen-deprived *A. mellifera*. 60 biological process GO terms were up-regulated in pollen starved bees in all three studies (Figure 5), and included processes related to stress response, ovary development, and sensory development (Additional file 9: Table S6). 19 biological process GO terms were down-regulated in starved bees in all three studies (Figure 5), including those related to lipid metabolism and biosynthesis (Additional file 10: Table S7). These results suggest that in bees fed pollen, most of the gene activity in the abdominal carcass tissue of nurses is concentrated on energy production and storage. In contrast, starved nurses exhibit very different expression patterns, including the up-regulation of stress response pathways and, in the case of sensory development, pathways that are likely correlated with a transition to precocious foraging.

## Conclusion

We found that substantial changes in gene expression occurred during early adult development and due to sub-optimal pollen intake in early adulthood. Diet-induced changes in gene transcription occurring in early age were largely a subset of those occurring at later age, but most signatures of starvation were only evident in older bees. The aging process itself differed when bees were deprived of pollen and the data are consistent with the hypothesis that poor diet causes normal age-related development to go awry. The preponderance of evidence from this study and other recent studies [18,19] indicates that adequate diet is critical to early worker development.

## Methods

### Bees and experimental manipulations

Three host colonies of *Apis mellifera* maintained for several months at the Carl Hayden Bee Research Center in Tucson, Arizona were used for dietary manipulations. The three host colonies were untreated (no miticides or antibiotics) 10 frame colonies with approximately 18,000 adults, 1 full frame face of brood, and 3.5 full frame faces of food stores. To collect newly emerged workers, sealed brood frames from approximately five colonies different from the host colonies used in the experiment were placed in a temperature-controlled room (approximately 34°C) and all adults that emerged in an 18 hour period were collected. These callow bees (≤ 18 hours of age) were marked using a small dot of non-toxic paint on their thorax and were added to the three host colonies, confined to each of two diets (a poor diet consisting of only stored honey or a rich diet of stored honey and bee bread) at a density of approximately one bee per cm^2^ (~40-60 bees per cage) using wire cages pushed into drawn comb over either diet. Bees were confined for 3 or 8 days in July and August of 2011. To sum, each of the three host colonies contained two dietary treatments, and the bees were sampled after being confined to that treatment for either 3 or 8 days, yielding three replicate (host) colonies for each diet (poor versus rich) by age (3d versus 8d) combination. Sampled bees were flash frozen in liquid nitrogen and maintained at -80°C until further processing.

### Hypopharyngeal gland (HG) measurements

Hypopharyngeal glands (HGs) of bees typically grow in preparation for nursing duties and this growth is impacted by the amount and source of dietary protein [10,41]. Our experimental design allowed trophallaxis (food sharing) with bees exterior to the treatment cage that had access to protein. To confirm that the bees caged over honey alone were indeed protein restricted compared to bees caged over the rich diet containing beebread and honey despite their access to other bees in the colony, the acini of HGs in bees aged 3d or 8d that were fed either diet were measured. HGs were dissected from approximately 5 bees per colony for each diet by age combination. 10 randomly selected acini per gland were visualized at 8X magnification. Acini area (mm^2^) was measured using the Leica Applications Suite v.3.8.0 software. Measurements were analyzed using a mixed model repeated measures ANOVA testing for the effect of diet, colony, age, the two-way interactions between colony and diet, age and diet, age and colony, and the three-way interaction between age, colony, and diet. Each acinus was a repeated measure taken on the same bee and a compound symmetry covariance structure was employed to model the correlation among measures taken on the same bee. A *post-hoc* analysis was performed using the Tukey HSD test to determine whether there were significant differences in acini area between diets (rich versus poor diet) for each age (3d and 8d old) and between ages for each diet.

### RNA extraction and library preparation

Abdominal carcasses were dissected from three bees per colony per treatment by removing their head and wings before making a dorsal incision through their abdominal cuticle, exposing the abdominal organs and forming a cup structure where RNAlater (Life Technologies) was added. After removing the digestive tract, the remaining tissue (i.e., the fat body, reduced reproductive organs, exoskeleton, trachea, and muscles) was separated from the thorax and this abdominal carcass was preserved at -80°C for subsequent RNA extraction. Three bees per host colony were pooled and total RNA was extracted using TriReagent (Invitrogen) according to the manufacturer’s specifications. Total RNA integrity was confirmed using Agilent’s 2100 Bioanalyzer.

Twelve mRNA sequencing (mRNA-Seq) libraries (2 ages × 2 diets × 3 replicate (host) colonies) were prepared using Illumina’s TruSeq RNA Sample Preparation Kit according to the manufacturer’s protocols. Briefly, poly-A containing mRNA was purified using poly-T oligo-attached magnetic beads and was subsequently fragmented and primed for first-strand cDNA synthesis with random hexamer primers. After degrading the RNA template used for first-strand cDNA synthesis, synthesis of the second cDNA strand followed, yielding a double-stranded cDNA molecule. Adapters were ligated to both ends of the double-stranded cDNA molecules and adapter-ligated cDNAs were enriched using 15 cycles of PCR using adapter-specific primers. The libraries were validated according to the manufacturer’s protocol and the approximately 350 bp fragments were isolated from a 6% Tris Base -Boric Acid-EDTA (TBE) PAGE gel and run through the Illumina sequencer for 2 × 100 cycles at a starting concentration of 12 pM per library.

### Analysis of paired end sequence data

Adaptor sequences were removed from each sequence and poor-quality reads were excluded using Trimmomatic [42] prior to the analysis of the ~150 base pair reads. The size distribution of the original transcripts for which sequence data was taken was first estimated by aligning the reads from each library separately to all publicly available *A. mellifera* RNA sequences listed in GenBank using the Burrows-Wheeler Aligner (BWA; [43]). Using the results from the BWA alignment, the expected mean and standard deviation of the inner distance between pairs was set for each library, and the reads were subsequently aligned to version 4.5 of the *A. mellifera* genome [44] with TopHat [45] version 1.4.0. The genome annotation contained NCBI reference sequence annotations and *ab initio* predictions based on the *A. mellifera* version 4.5 genome [46]. First, we analyzed a full model to determine whether the effects of age, diet, and the interaction between age and diet significantly impacted gene expression using the edgeR package [47] and a Benjamini-Hochberg correction for multiple testing [48] at a 5% false discovery rate. To test whether the effect of diet was different for 3d old versus 8d old bees, the effect of diet was investigated for each age separately. To test whether the effects of aging on gene expression differed with respect to diet, the effect of aging was studied separately for bees fed pollen and those that were not fed pollen.

Statistical analyses of differential expression yielded a corrected significance value for each exon that mapped to each mRNA transcript in the *Apis mellifera* version 4.5 genome annotation [44]. If ≥2 exons mapped to the same transcript, we reasoned that the entire mRNA transcript (irrespective of isoform) was differentially expressed. Therefore, single exon exon transcripts (i.e., the only significantly differentially expressed exon mapping to an annotated transcript) were eliminated from further analyses. This approach was conservative because it eliminated the occurrence of false positives but came at a cost because it also removed single-exon transcripts from further analyses. In addition, we did not analyze alternative splicing events, which were beyond the scope of the study.

The significantly differentially expressed transcripts (containing ≥2 exons) were subjected to further characterization. *Drosophila melanogaster* genes orthologous to the differentially expressed *A. mellifera* transcripts were identified by searching for reciprocal best BLAST hits between *A. mellifera* mRNA sequences and *D. melanogaster* proteins. A FlyBase gene ID (FBgn) [49] was assigned to all *A. mellifera* transcripts where a *D. melanogaster* orthologue was identified. GeneCoDis3 [50-52] was then employed to find enriched biological process gene ontology (BP GO) terms and gene annotation co-occurrence clusters (i.e., annotation clusters) that were significantly associated with the differentially expressed orthologues [50-52]. In addition to single BP GO annotations, annotation clusters represented significant relationships among biological process, molecular function, InterPro protein domain, and KEGG pathway annotations that were associated with the differentially expressed orthologoues [50-52]. A reference background list of all *D. melanogaster* genes orthologous to all *A. mellifera* RNAs was used to determine significant enrichment of BP GO terms and annotation clusters using a chi-square (*X*^2^) test corrected for multiple testing [48]. Significantly enriched BP GO terms that contained only one orthologue were discarded.

The annotated transcripts (containing ≥2 exons) and orthologues that exhibited differential expression were compared. To address the hypothesis that diet and early adult development affect *A. mellifera* gene expression and physiology, annotated transcripts that increased or decreased due to starvation or as individuals aged from 3d to 8d old were compared and biological processes and annotation clusters corresponding to differentially expressed orthologues were analyzed for significant enrichment. To address the hypothesis that starvation affects younger and older workers differently, comparisons were made between annotated transcripts and orthologues that were affected by diet at either 3d or 8d of age. Venn Diagrams were constructed using the annotated transcripts that were differentially expressed due to diet at 3d and those that were differentially expressed due to diet at 8d. To address the last hypothesis that development and aging are affected by diet, comparisons were made between annotated transcripts and orthologues that changed with age for bees fed either a rich diet or those fed a poor diet. Venn Diagrams were constructed using the annotated transcripts that were differentially expressed due to age for the rich diet and those that were differentially expressed due to age in bees deprived of pollen.

### rt-PCR validation of gene expression changes due to diet

To assess the predictive power of the mRNA-seq analyses, we measured the expression of six genes – vermiform (XM623720), cdc42 (XM_394608), E75 (NM_001080110), GTP-binding protein 10 (XM_396976), vitellogenin (NM_001011578), glucocerebrosidase transcript variant 1 (XM_393207; Additional file 11: Table S8) – that were significantly differentially expressed based on the previous mRNA-seq experiment. Fat bodies of 8d old bees from three replicate host colonies (*N*=3) were used. These bees were not used in the previous mRNA-seq experiment, but the colonies they came from shared similar population sizes, age distributions, and distribution of food stores to the three colonies used in the mRNA-seq experiments and to each other. Dietary manipulations, fat body dissections, and RNA isolations were as described above except RNA integrity was not assayed. To test that the dietary manipulations were successful, HGs were dissected and acini were measured in the manner described above. RNA was isolated from three pooled fat bodies per colony for each age and for each dietary treatment, yielding a total of six samples (3 host colonies x 2 diets). Isolated RNA was subjected to a DNase treatment (Ambion) and the lack of genomic DNA contamination was verified by performing a PCR on the RNA with *A. mellifera* actin (AB023025.1; Additional file 11: Table S8) and the following cycling conditions: 3 minutes at 94°C, 35 cycles of 45 seconds at 94°C, 45 seconds at 57°C, and 45 seconds at 72°C, and a final extension of 72°C for 10 minutes. cDNA was synthesized using the iScript cDNA synthesis kit (Bio-Rad) according to the manufacturer’s protocol. The amplification efficiencies of all genes ranged between 95% and 105%. For each gene, a two-step qPCR was performed on the cDNA using SsoFast EvaGreen Supermix (Bio-Rad) according to the manufacturer’s protocol and the following cycling conditions (annealing temperatures, *n*, are listed in Additional file 11: Table S8): 3 minutes at 95°C, 45 cycles of 10 seconds at 95°C and 10 seconds at *n*°C, followed by a melt curve from 55°C to 95°C to confirm the lack of contamination and/or non-specific amplification. For each of the three colonies per diet, the threshold cycle (C_T_) values from three technical replicates were averaged for each target gene and two standards: *A. mellifera* actin (AB023025.1) and GAPDH (XM_393605; Additional file 11: Table S8). The average C_T_ values of each target gene were normalized relative to the mean C_T_ of each standard separately, accounting for primer amplification efficiencies [53]. Significant differences between the diets were determined using a Mann–Whitney/Wilcoxon test [54] and results were significant only if results were different from one for both of standards (actin and GAPDH).

### Availability of supporting data

Additional file 6: Table S4 supporting the results of this article is available in the Dryad Digital Repository http://doi.org/10.5061/dryad.pg2kb. All reads were submitted to the NCBI Sequence Read Archive under accession number SRA056350.

## Competing interests

The authors declare that they have no competing interests.

## Authors’ contributions

VC-H and KEA conceived of the experiments. VC-H designed the experiments. VC-H, BMJ, AW, and MRS performed the experiments. VC-H analyzed the data. KEA contributed reagents and materials. VC-H wrote the paper and all authors approved of the final manuscript.

## Supplementary Material

Additional file 1: Table S1 Biological process gene ontology (GO) terms that showed higher expression in starved bees compared to bees fed pollen in both Ament *et al*. [19] and Alaux *et al*. [18]. Click here for file

Additional file 2: Table S2 Biological process gene ontology (GO) terms that showed reduced expression in starved bees compared to bees fed pollen in both Ament *et al*. [19] and Alaux *et al*. [18]. Click here for file

Additional file 3: Figure S1 The core set of biological process impacted by diet in previous studies. Biological process gene ontology (GO) terms that showed similar expression patterns in starved bees compared to bees fed pollen as determined by Ament *et al*. [19] and Alaux *et al*. [18]. Click here for file

Additional file 4: Figure S2 Hypopharyngeal gland (HG) sizes of well-fed and nurse workers deprived of pollen, separated by host colony. HG size, as measured by HG acinus size (mm^2^), is presented for bees raised for either 3 days (3d) or 8 days (8d) and fed a diet of honey alone (H) or honey and pollen (HP) for colonies A, B, and C. Error bars represent standard error for the mean acinus size for the five individuals tested for each diet x age combination. Significant results of a *post-hoc* Tukey-Kramer test on the mean acinus sizes for each diet by age combination are presented. Click here for file

Additional file 5: Table S3 Summary of sequencing effort and read alignment to the *Apis mellifera* genome. Click here for file

Additional file 6: Table S4 List of all differentially expressed transcripts. Data presented include (from left to right) the genome accession number (column A) and start and end positions (columns B and C) where that differentially expressed exon is located on the *A. mellifera* genome, the size of the exon (column D), the exon annotation in NCBI’s GNOMON annotation (column E; the number after the colon indicates the exon number), the *D. melanogaster* orthologue matching the transcript (column F; FBgns indicate the FlyBase ID), the accession number and description for the mRNA at that genomic location (columns G and H), and whether the full model yielded a significant result for the main effect of diet (column I) or age (column K) after a correction for multiple tests. Significance of pairwise contrasts between diets at 3 days (column M) and 8 days (column O), and between young and old bees fed a poor diet (column Q) or a rich diet containing beebread (column S) are presented. Where the effect was significant, the direction and magnitude of the expression change between diets (column J), between ages (column L), between diets in only young bees (column N), between diets in older bees (column P), between ages in bees fed a poor diet (column R) and between ages in bees fed a rich diet (column T) are presented and were calculated using the expression estimates in columns U through AF. Columns U through AF include the fitted expression estimates for each exon and for each host colony x age x diet combination following a generalized linear model analysis of the raw expression values (edgeR; see text and [47,55]). Click here for file

Additional file 7: Table S5 Exon-based analysis of the number of genes changing with respect to diet or age. Click here for file

Additional file 8: Figure S3 Comparisons of the magnitude of age-associated (A) up- or (B) down-regulation in bees fed or deprived of pollen. For each exon that was significantly affected by age in bees fed a poor diet (deprived of pollen) or a rich diet (containing pollen), the average expression among host colonies was calculated for each age by diet combination. The magnitude of age-related change was then calculated by dividing the values obtained in young bees by the values obtained in old bees separately for each diet. This yielded estimates of up-regulation with age (panel A, values are greater than one because expression was higher in old bees compared to young bees) and down-regulation with age (panel B, values are less than one because expression was higher in young bees compared to old bees) for bees fed each type of diet. Means of these values across exons are presented along with the standard error around this mean. Click here for file

Additional file 9: Table S6 Biological process gene ontology (GO) terms that were up-regulated in starved bees compared to bees fed pollen in the present study, Ament *et al*. [19], and Alaux *et al*. [18]. Click here for file

Additional file 10: Table S7 Biological process gene ontology (GO) terms with reduced expression in starved bees compared to bees fed pollen in the present study, Ament *et al*. [19], and Alaux *et al*. [18]. Click here for file

Additional file 11: Table S8 List of rt-PCR primers used in this study. Click here for file
