# Supplementary figures and images for: Transcriptional markers of sub-optimal nutrition in developing Apis mellifera nurse workers
Source: BMC Genomics. 2014 Feb 15;15:134. doi: 10.1186/1471-2164-15-134 (PMC3933195; doi:10.1186/1471-2164-15-134)

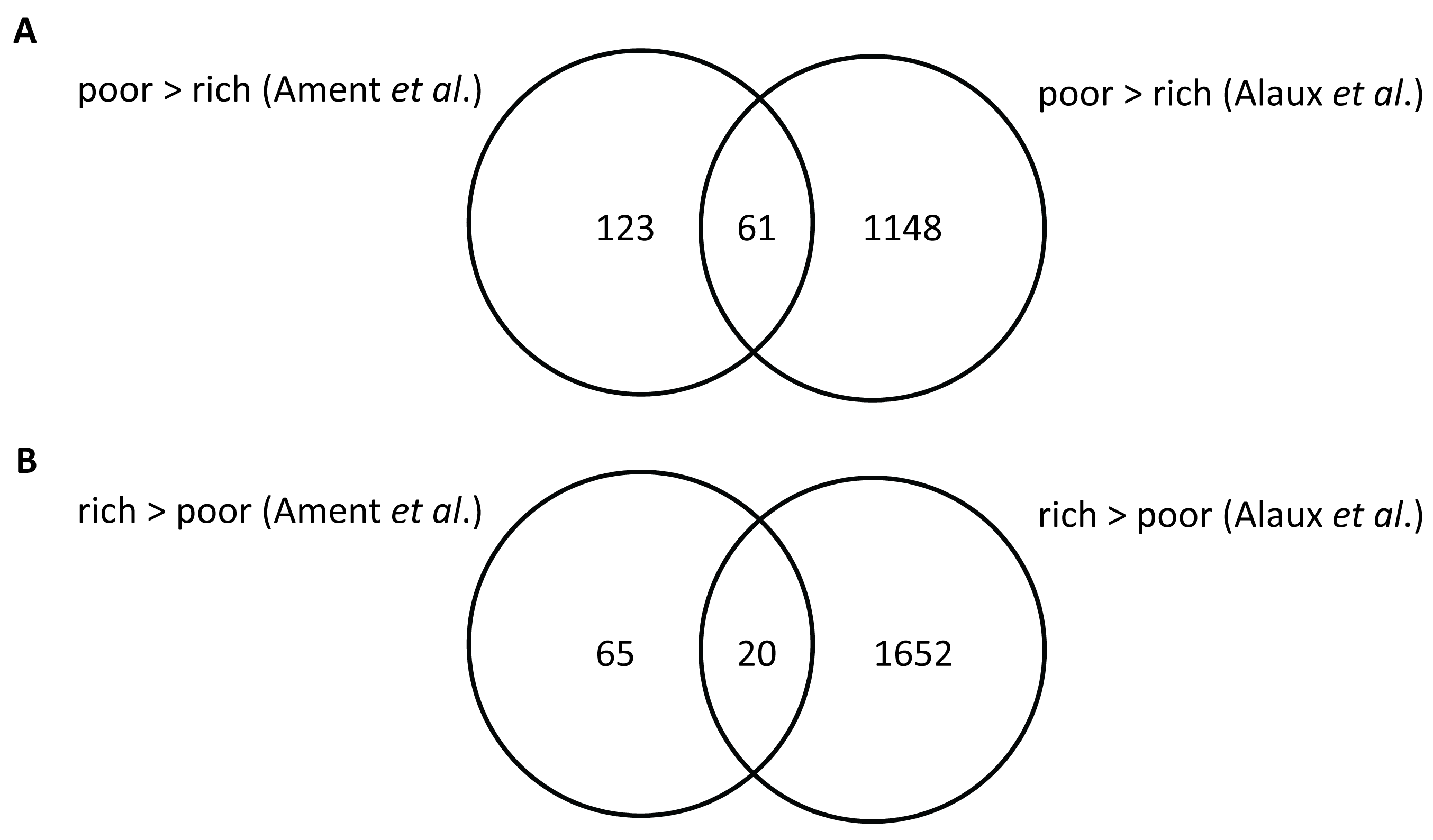

Supplement: Additional file 3: Figure S1 — The core set of biological process impacted by diet in previous studies. Biological process gene ontology (GO) terms that showed similar expression patterns in starved bees compared to bees fed pollen as determined by Ament et al. [19] and Alaux et al. [18]. [file 1471-2164-15-134-S3.tiff]

## Slide 1
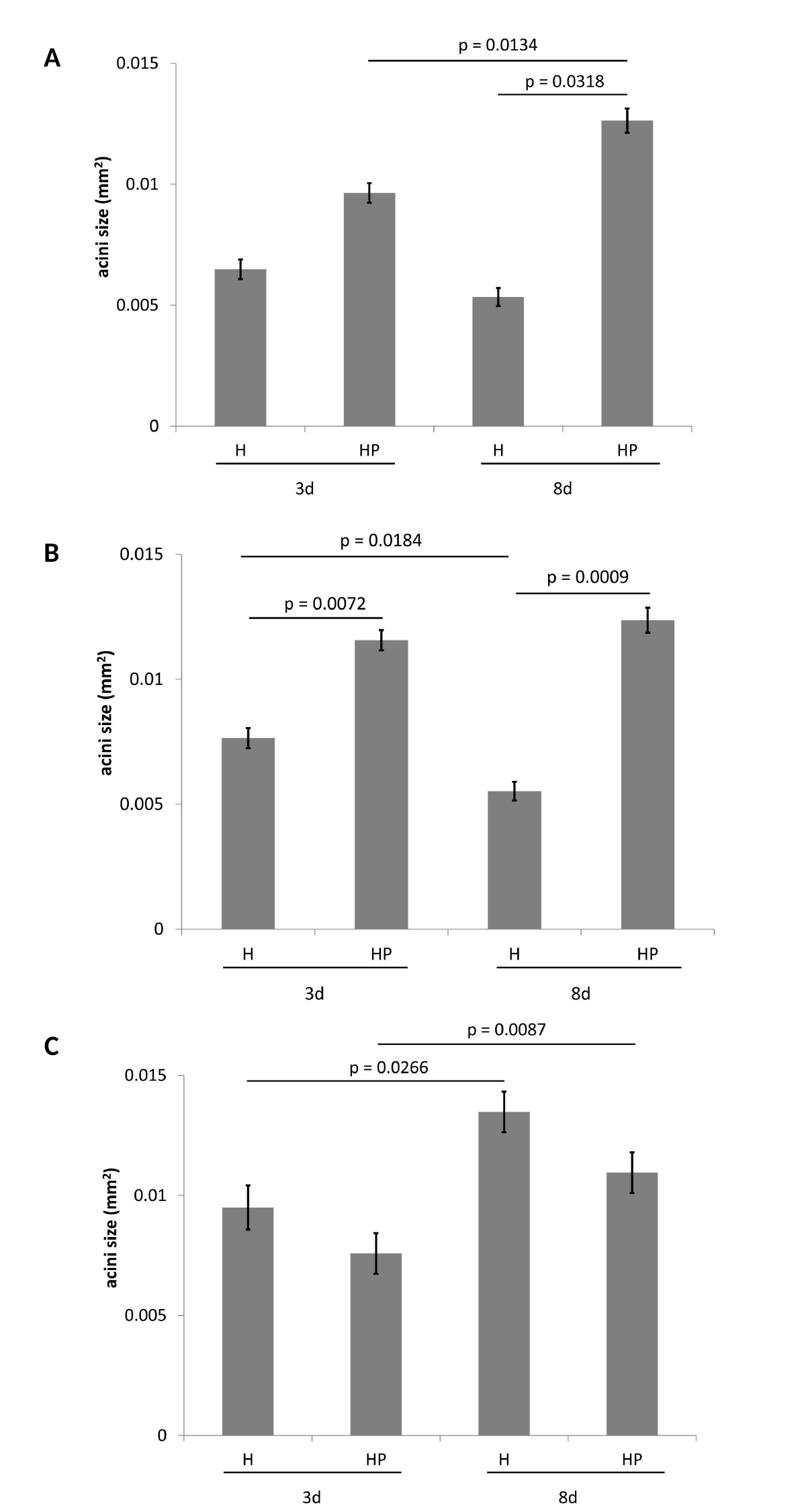

A
B
C

Supplement: Additional file 4: Figure S2 — Hypopharyngeal gland (HG) sizes of well-fed and nurse workers deprived of pollen, separated by host colony. HG size, as measured by HG acinus size (mm2), is presented for bees raised for either 3 days (3d) or 8 days (8d) and fed a diet of honey alone (H) or honey and pollen (HP) for colonies A, B, and C. Error bars represent standard error for the mean acinus size for the five individuals tested for each diet x age combination. Significant results of a post-hoc Tukey-Kramer test on the mean acinus sizes for each diet by age combination are presented. [file 1471-2164-15-134-S4.pptx]

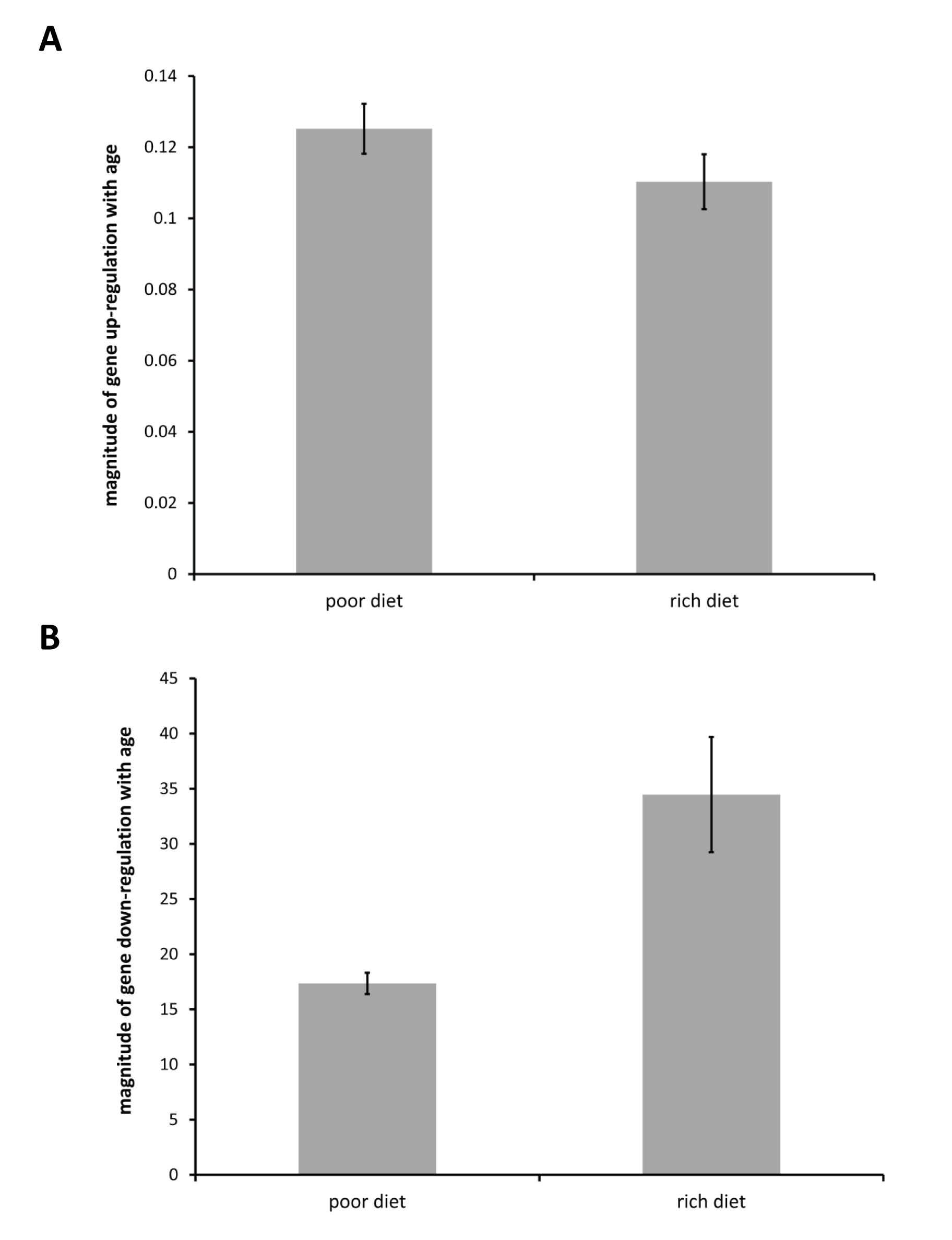

Supplement: Additional file 8: Figure S3 — Comparisons of the magnitude of age-associated (A) up- or (B) down-regulation in bees fed or deprived of pollen. For each exon that was significantly affected by age in bees fed a poor diet (deprived of pollen) or a rich diet (containing pollen), the average expression among host colonies was calculated for each age by diet combination. The magnitude of age-related change was then calculated by dividing the values obtained in young bees by the values obtained in old bees separately for each diet. This yielded estimates of up-regulation with age (panel A, values are greater than one because expression was higher in old bees compared to young bees) and down-regulation with age (panel B, values are less than one because expression was higher in young bees compared to old bees) for bees fed each type of diet. Means of these values across exons are presented along with the standard error around this mean. [file 1471-2164-15-134-S8.tiff]
