# Supplementary material for: Transcriptional markers of sub-optimal nutrition in developing Apis mellifera nurse workers
Source: BMC Genomics. 2014 Feb 15;15:134. doi: 10.1186/1471-2164-15-134 (PMC3933195; doi:10.1186/1471-2164-15-134)
Supplement: Additional file 5: Table S3 — Summary of sequencing effort and read alignment to the Apis mellifera genome. [file 1471-2164-15-134-S5.docx]

Table S3. Summary of sequencing effort and read alignment to the *A. mellifera* genome.

| Library Information | | | | | |
| --- | --- | --- | --- | --- | --- |
| Age (days) | Diet | Colony | Reads in library | Total Mapped ^A^ | % mapped in library ^B^ |
| 3 | Honey | A | 8.82 | 6.29 | 71.3 |
|  |  | B | 9.70 | 5.65 | 58.2 |
|  |  | C | 25.7 | 20.39 | 79.3 |
| 3 | Honey and Pollen | A | 8.49 | 4.80 | 56.6 |
|  |  | B | 12.12 | 8.00 | 66.0 |
|  |  | C | 11.16 | 8.84 | 79.2 |
| 8 | Honey | A | 15.44 | 11.42 | 74.0 |
|  |  | B | 10.57 | 6.86 | 64.8 |
|  |  | C | 8.60 | 5.77 | 67.1 |
| 8 | Honey and Pollen | A | 12.01 | 7.44 | 62.0 |
|  |  | B | 16.01 | 10.42 | 65.1 |
|  |  | C | 10.78 | 7.10 | 65.9 |
|  | Total |  | 149.4 | 102.98 | 68.9 |

^A^ Millions of paired end reads.

^B^ Millions of paired end reads mapped to the *A. mellifera* genome.

^C^ Percent of total paired-end reads successfully mapped to the *A. mellifera* genome.
