# Supplementary material for: Transcriptional markers of sub-optimal nutrition in developing Apis mellifera nurse workers
Source: BMC Genomics. 2014 Feb 15;15:134. doi: 10.1186/1471-2164-15-134 (PMC3933195; doi:10.1186/1471-2164-15-134)
Supplement: Additional file 7: Table S5 — Exon-based analysis of the number of genes changing with respect to diet or age. [file 1471-2164-15-134-S7.docx]

Table S5. Exon-based analysis of expression change due to diet or age (not genes as in Table 2).

| factor | constant | exons changed^A^ | increased^A^ | decreased^A^ | % exons decreased^B^ | Difference in % decrease? | |
| --- | --- | --- | --- | --- | --- | --- | --- |
|  |  |  |  |  |  | *Χ*^2^ | *p* |
| starvation | 3d | 386 | 26 | 360 | 93.3 | 144.83 | <0.0001 |
|  | 8d | 66811 | 24314 | 42497 | 63.6 |  |  |
| aging | poor diet | 256 | 72 | 184 | 71.9 | 22.59 | <0.0001 |
|  | rich diet | 185 | 94 | 91 | 49.2 |  |  |

^A^ For exons where ≥ 2 exons were differentially expressed in one gene. Genes represented by only one differentially expressed exon were excluded.

^B^ Of the transcripts that differed due to each factor combination.
