# Supplementary material for: Transcriptional markers of sub-optimal nutrition in developing Apis mellifera nurse workers
Source: BMC Genomics. 2014 Feb 15;15:134. doi: 10.1186/1471-2164-15-134 (PMC3933195; doi:10.1186/1471-2164-15-134)
Supplement: Additional file 9: Table S6 — Biological process gene ontology (GO) terms that were up-regulated in starved bees compared to bees fed pollen in the present study, Ament et al. [19], and Alaux et al. [18]. [file 1471-2164-15-134-S9.pdf]

Table S6. Biological process gene ontology (GO) terms that were up-regulated in bees deprived of pollen in Alaux *et al* . (2011), Ament *et al* . (2011), and the present study.

BP GO term	description
GO:0000003	reproduction
GO:0000226	microtubule cytoskeleton organization
GO:0000902	cell morphogenesis
GO:0000910	cytokinesis
GO:0001700	embryonic development via the syncytial blastoderm
GO:0001745	compound eye morphogenesis
GO:0002165	instar larval or pupal development
GO:0006139	nucleobase-containing compound metabolic process
GO:0006259	DNA metabolic process
GO:0006325	chromatin organization
GO:0006351	transcription, DNA-templated
GO:0006355	regulation of transcription, DNA-templated
GO:0006357	regulation of transcription from RNA polymerase II promoter
GO:0006366	transcription from RNA polymerase II promoter
GO:0006396	RNA processing
GO:0006397	mRNA processing
GO:0006464	cellular protein modification process
GO:0006468	protein phosphorylation
GO:0007010	cytoskeleton organization
GO:0007049	cell cycle
GO:0007067	mitosis
GO:0007154	cell communication
GO:0007163	establishment or maintenance of cell polarity
GO:0007165	signal transduction
GO:0007264	small GTPase mediated signal transduction
GO:0007265	Ras protein signal transduction
GO:0007275	multicellular organismal development
GO:0007399	nervous system development
GO:0007409	axonogenesis
GO:0007411	axon guidance
GO:0007423	sensory organ development
GO:0007444	imaginal disc development
GO:0007455	eye-antennal disc morphogenesis
GO:0007476	imaginal disc-derived wing morphogenesis
GO:0007498	mesoderm development
GO:0007517	muscle organ development
GO:0007552	metamorphosis
GO:0008283	cell proliferation
GO:0008361	regulation of cell size
GO:0009266	response to temperature stimulus
GO:0009408	response to heat
GO:0009790	embryo development
GO:0009792	embryo development ending in birth or egg hatching

GO:0009888	tissue development
GO:0009987	cellular process
GO:0010468	regulation of gene expression
GO:0016055	Wnt signaling pathway
GO:0019222	regulation of metabolic process
GO:0022008	neurogenesis
GO:0030036	actin cytoskeleton organization
GO:0030154	cell differentiation
GO:0030707	ovarian follicle cell development
GO:0035220	wing disc development
GO:0040007	growth
GO:0042127	regulation of cell proliferation
GO:0048477	oogenesis
GO:0048749	compound eye development
GO:0048812	neuron projection morphogenesis
GO:0051276	chromosome organization
GO:0051726	regulation of cell cycle
