# Supplementary material for: Transcriptional markers of sub-optimal nutrition in developing Apis mellifera nurse workers
Source: BMC Genomics. 2014 Feb 15;15:134. doi: 10.1186/1471-2164-15-134 (PMC3933195; doi:10.1186/1471-2164-15-134)
Supplement: Additional file 10: Table S7 — Biological process gene ontology (GO) terms with reduced expression in starved bees compared to bees fed pollen in the present study, Ament et al. [19], and Alaux et al. [18]. [file 1471-2164-15-134-S10.pdf]

Table S7. Biological process gene ontology (GO) terms that were up-regulated in bees fed pollen in Alaux *et al* . (2011), Ament *et al* . (2011), and the present study.

BP GO term	description
GO:0006099	tricarboxylic acid cycle
GO:0006119	oxidative phosphorylation
GO:0006413	translational initiation
GO:0006520	cellular amino acid metabolic process
GO:0006626	protein targeting to mitochondrion
GO:0006629	lipid metabolic process
GO:0006631	fatty acid metabolic process
GO:0006635	fatty acid beta-oxidation
GO:0006807	nitrogen compound metabolic process
GO:0007005	mitochondrion organization
GO:0008152	metabolic process
GO:0009058	biosynthetic process
GO:0009156	ribonucleoside monophosphate biosynthetic process
GO:0009165	nucleotide biosynthetic process
GO:0015986	ATP synthesis coupled proton transport
GO:0015992	proton transport
GO:0044237	cellular metabolic process
GO:0045454	cell redox homeostasis
GO:0046034	ATP metabolic process
