# Supplementary material for: Transcriptional markers of sub-optimal nutrition in developing Apis mellifera nurse workers
Source: BMC Genomics. 2014 Feb 15;15:134. doi: 10.1186/1471-2164-15-134 (PMC3933195; doi:10.1186/1471-2164-15-134)
Supplement: Additional file 11: Table S8 — List of rt-PCR primers used in this study. [file 1471-2164-15-134-S11.docx]

Table S8. rtPCR primers used in this study.

| Gene | Accession | Forward primer | Reverse primer | *n*^A^ | expected change |
| --- | --- | --- | --- | --- | --- |
| vermiform | XM_623720 | GCTCCTTCTTGGATCCCTATTT | GCTTGCTTTGTCGCATCTTAC | 54 | rich > poor |
| Cdc42 | XM_394608 | TGGGTACCCGAAATAACACATC | TGCGTAAGAGCACTACATTCTAC | 52 | rich > poor |
| E75 | NM_001080110 | CCAGGATTCTGGCTGCTATG | GCGGTATAGTTAGGCGTTTCTC | 52 | rich > poor |
| GTP-binding protein 10 | XM_396976 | TGGAGGTACAGGTGGTTCAGGATT | GGAACTTTCACTTCCATTTCCTGC | 55 | rich > poor |
| vitellogenin | NM_001011578 | CCCACGTTGATCTCCAACTAC | CCGCTTGTCTTGGTCAACTTT | 58 | rich > poor |
| Glucocerebrosidase transcript variant 1 | XM_393207 | TTTACCGACTCGGCGGGAATGAAT | ATGTTGCAACGTCGCATCGTCATC | 55 | poor > rich |
| GAPDH | XM_393605 | GATGCACCCATGTTTGTTTG | TTTGCAGAAGGTGCATCAAC |  |  |
| Actin | NM_001185146 | TGCCAACACTGTCCTTTCTG | AGAATTGACCCACCAATCCA |  |  |

^A^ Transcript and exon in GNOMON annotation corresponding to rtPCR amplicon.

^B^ Annealing temperature of the primer, noted as *n* in the PCR protocol outlined in the Materials and Methods.

^C^ Expected direction of expression change in bees fed either a rich or poor diet. Actin and GAPDH served as reference genes.
